# P-1190. Viral Codetection in Infants with Respiratory Syncytial Virus (RSV) Infection Is Associated with Decreased Viral Loads, Reduced Mucosal Cytokine Concentrations and Longer Hospitalization

**DOI:** 10.1093/ofid/ofae631.1374

**Published:** 2025-01-29

**Authors:** Irene Tzovara, Jeanette Taveras, Cristina Garcia-Maurino, Helena Brenes-Chacon, Sara Mertz, Fang Ye, Zhaohui Xu, Diego R Hijano, Octavio Ramilo, Asuncion Mejias

**Affiliations:** Aghia Sophia Children's Hospital, Galatsi, Attiki, Greece; Nationwide Children's Hospital, Columbus, Ohio; N/A, Columbus, Ohio; Caja Costarricense del Seguro Social, San Jose, San Jose, Costa Rica; The Research Institute at Nationwide Children's Hospital, Columbus, Ohio; Nationwide Childrens Hospital, Columbus, Ohio; St. Jude Children's Research Hospital, Memphis, TN; St. Jude Children's Research Hospital, Memphis, TN; St. Jude Children's Research Hospital, Memphis, TN; St Jude Children's Research Hospital, Memphis, TN

## Abstract

**Background:**

Studies have analyzed the contribution of host and viral factors to RSV disease pathogenesis and severity in children, but the role of RSV/viral co-detection is poorly understood. We investigated the relationship between viral co-detection, RSV viral loads, mucosal cytokine concentrations and disease severity in young children with RSV acute respiratory infection (ARI).

**Figure:**
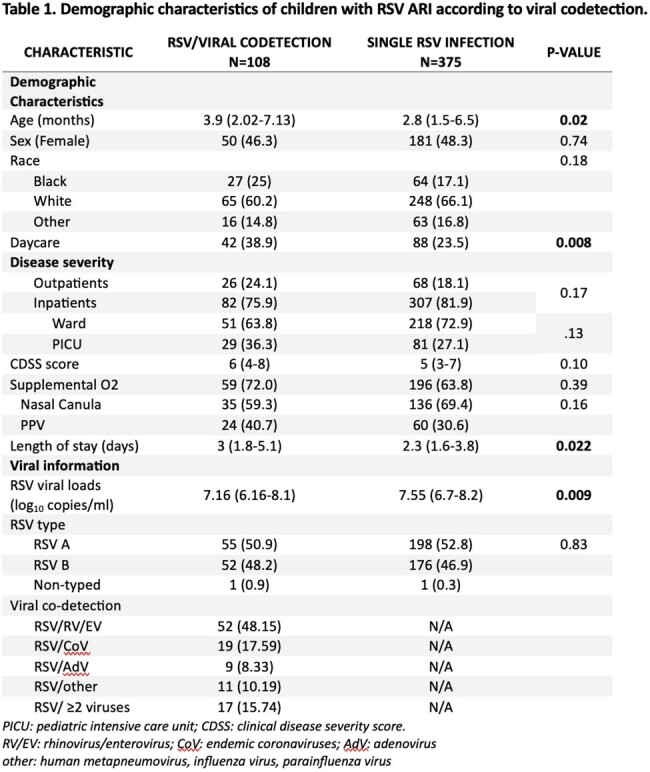

**Methods:**

We enrolled a convenience sample of previously healthy children < 2 years of age with RSV ARI that were either hospitalized or managed as outpatients at a large pediatric hospital. Nasopharyngeal samples were obtained to assess RSV viral loads by rt-PCR, viral codetection using a multiplex PCR assay, and mucosal cytokine concentrations. We assessed the impact of viral co-detection on RSV viral loads, mucosal cytokine concentrations, and clinical outcomes stratified by age (0-6 and >6-24 months).

**Figure 1.**
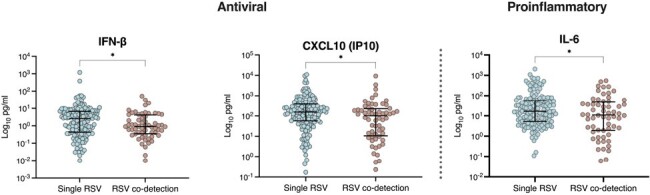
Mucosal cytokine concentrations according to single RSV detection versus RSV/viral co-detection .

The Y axis depicts individual concentrations of mucosal antiviral cytokines (IFN-β and CXCL-10) and of IL-6 expressed as log10 pg/ml, and the x axis the two study groups: single RSV infections and RSV/viral co-detections. Comparisons between groups were made with Mann-Whitney test and statistical significance was set at p< 0.05.

**Results:**

From February 2013 to March 2020, 483 children with RSV ARI were enrolled (inpatients, n=383; outpatients, n=95). Of those, 108 (22.4%) had viral co-detection. Rhinovirus/Enterovirus (RV/EV) followed by endemic coronaviruses (CoV) were the most common co-detected viruses. Median age was 3.9 months for infants with RSV/viral co-detection vs 2.8 months for with single RSV ARI (p=0.02); with no significant differences in other demographic characteristics (Table 1). RSV loads were higher in single RSV vs RSV/viral co-detection (7.55 vs 7.16 log_10_ copies/ml, p=0.0009), particularly in infants 0-6 months. Mucosal concentrations of IL-6, CXCL-10, and IFN-β were significantly higher in children with single RSV vs RSV/viral co-detection (Figure 1). Analysis by age showed significant differences of IL-6 and CXCL-10 concentrations in infants 0-6 months, but not in older children. There were no significant differences with respect to need for hospitalization, clinical disease severity or PICU care in infants with single RSV vs RSV/viral codetections, but RSV/viral co-detection was associated with longer hospitalizations (3 vs 2.3 days respectively, p=0.022).

**Conclusion:**

Concomitant detection of other respiratory viruses in children with RSV ARI was common and associated with reduced RSV viral loads, decreased mucosal proinflammatory and antiviral cytokines, and longer length of stay.

**Disclosures:**

**Diego R. Hijano, MD, MSc**, FDA: Grant/Research Support|Merck: Grant/Research Support|National Institute of Health: Grant/Research Support **Octavio Ramilo, MD**, AstraZeneca: Honoraria|Bill & Melinda Gates Foundation: Grant/Research Support|Merck: Advisor/Consultant|Merck: Grant/Research Support|Merck: Honoraria|NIH/NIAID: Board Member|NIH/NIAID: Grant/Research Support|NIH/NICHD: Grant/Research Support|Pfizer: Advisor/Consultant|Pfizer: Honoraria|Sanofi: Advisor/Consultant|Sanofi: Honoraria **Asuncion Mejias, MD, PhD, MsCS**, Astra-Zeneca: Advisor/Consultant|Astra-Zeneca: Honoraria|Enanta: Advisor/Consultant|Janssen: Advisor/Consultant|Janssen: Grant/Research Support|Merck: Advisor/Consultant|Merck: Grant/Research Support|Moderna: Advisor/Consultant|Pfizer: Advisor/Consultant|Pfizer: Honoraria|Sanofi-Pasteur: Advisor/Consultant|Sanofi-Pasteur: Honoraria

